# Refining the risk of HTLV-1-associated myelopathy in people living with HTLV-1: identification of a HAM-like phenotype in a proportion of asymptomatic carriers

**DOI:** 10.1007/s13365-022-01088-x

**Published:** 2022-07-30

**Authors:** Daniel Harding, Carolina Rosadas, Sandra Maria Tsoti, Amanda Heslegrave, Molly Stewart, Peter Kelleher, Henrik Zetterberg, Graham P. Taylor, Divya Dhasmana

**Affiliations:** 1 Section of Virology, Department of Infectious Disease, Imperial College London, London, W2 1PG UK; 2 UK Dementia Research Institute at UCL, London, UK; 3 Department of Neurodegenerative Disease, UCL Institute of Neurology, London, UK; 4 Department of Infection and Immunity Sciences, North West London Pathology, Charing Cross Hospital, London, UK; 5 Section of Immunology of Infection, Department of Infectious Disease, Imperial College London, London, UK; 6 Clinical Neurochemistry Laboratory, Sahlgrenska University Hospital, Mölndal, Sweden; 7 Department of Psychiatry and Neurochemistry, Institute of Neuroscience and Physiology, Sahlgrenska Academy at the University of Gothenburg, Mölndal, Sweden; 8 Hong Kong Centre for Neurodegenerative Diseases, Hong Kong, China; 9 National Centre for Human Retrovirology, Imperial College Healthcare NHS Trust, London, W2 1NY UK

**Keywords:** HTLV-1, HTLV-1-associated myelopathy, Proviral load, T-cell activation markers, β_2_ microglobulin

## Abstract

Up to 3.8% of human T-lymphotropic virus type-1 (HTLV-1)-infected asymptomatic carriers (AC) eventually develop HTLV-1-associated myelopathy (HAM). HAM occurs in patients with high (> 1%) HTLV proviral load (PVL). However, this cut-off includes more than 50% of ACs and therefore the risk needs to be refined. As HAM is additionally characterised by an inflammatory response to HTLV-1, markers of T cell activation (TCA), β_2_-microglobulin (β_2_M) and neuronal damage were accessed for the identification of ACs at high risk of HAM. Retrospective analysis of cross-sectional and longitudinal routine clinical data examining differences in TCA (CD4/CD25, CD4/HLA-DR, CD8/CD25 & CD8/HLA-DR), β_2_M and neurofilament light (NfL) in plasma in ACs with high or low PVL and patients with HAM. Comparison between 74 low PVL ACs, 84 high PVL ACs and 58 patients with HAM revealed a significant, stepwise, increase in TCA and β_2_M. Construction of receiver operating characteristic (ROC) curves for each of these blood tests generated a profile that correctly identifies 88% of patients with HAM along with 6% of ACs. The 10 ACs with this ‘HAM-like’ profile had increased levels of NfL in plasma and two developed myelopathy during follow-up, compared to none of the 148 without this viral-immune-phenotype. A viral-immuno-phenotype resembling that seen in patients with HAM identifies asymptomatic carriers who are at increased risk of developing HAM and have markers of subclinical neuronal damage.

**Supplementary Information:**

The online version contains supplementary material available at 10.1007/s13365-022-01088-x.

## Introduction

Human T-lymphotropic virus type-1 (HTLV-1) is a human retrovirus estimated to infect 5–10 million individuals globally, with the highest prevalence occurring in South-west Japan, the Caribbean, South America and sub-Saharan Africa (Gessain and Cassar 2012).

Following permanent host cell genome integration, predominantly in CD4 + T-lymphocytes, HTLV-1 most frequently causes adult T-cell leukaemia/lymphoma (ATL) (Hinuma et al. 1981) or HTLV-1-associated myelopathy (HAM), formerly tropical spastic paraparesis (Gessain et al. 1985; Osame et al. 1986). Other known HTLV-1-associated inflammatory conditions include infective dermatitis (LaGrenade et al. 1990), polymyositis (Morgan et al. 1989), bronchiectasis (Einsiedel et al. 2012), uveitis (Mochizuki et al. 1992), autoimmune thyroiditis (Kawai et al. 1992) and Sjögren’s syndrome (Nakamura et al. 1997). The estimates of cumulative lifetime risk for HAM for HTLV-1 carriers range from 0.3 (Kaplan et al. 1990) to 3.8% (Orland et al. 2003). HAM is a chronic progressive myelopathy, with a theorised pathophysiology involving expanding populations of HTLV-1-infected CD4 + and HTLV-1-specific CD8 + T lymphocytes more capably migrating into the spinal cord, at which point, their interaction with one another generates neurotoxic cytokines such as IFN-γ and TNF-α, inadvertently initiating chronic myelitis (Höllsberg 1997).

The factor characterising an asymptomatic carrier’s (AC) risk of developing HAM with the largest body of evidence is HTLV-1 proviral load (PVL), commonly quantified as the number of HTLV-1 proviral copies per 100 peripheral blood mononuclear cells (PBMC) and expressed as a percentage. Association between HAM and PVL has been demonstrated in multiple studies (Nagai et al. 2014; Brito et al. 2012; Grassi et al. 2011; Taylor et al. 1999) with an exponential increase in HAM reported above 1% (Nagai et al. 2014). HTLV-1 PVL remains stable over several years regardless of disease expression (Demontis et al. 2012; Kwaan et al. 2006). However, the use of PVL to determine HAM risk is limited by the significant proportion of ACs with a PVL above 1%, which in the UK cohort was 57% (Demontis et al. 2012). Even when based on the highest lifetime risk estimate (Orland et al. 2003), prognostic use of PVL generates a false positive rate above 90%. Thus, additional markers are required to refine the measurement of HAM risk in ACs. β_2_-microglobulin (β_2_M) was identified as a marker of both HAM and disease activity in a proteomic study (Kirk et al. 2011) whilst activation of T-cells has been observed in patients with HTLV-1-associated myelopathy (Ijichi et al. 1989). One study examining T cell activation (TCA) markers in 20 ACs showed that high PVL is associated with T cell activation (Coutinho et al. 2014), but these markers have yet to be comprehensively investigated in patients with HAM and ACs with a broad range of PVL.

The trigger(s) for HAM is not known but given that the onset is frequently indolent, we hypothesised that a subset of ACs would have a viral and immune phenotype similar to that of patients with HAM and that this viral-immune-phenotype would predict the development of HAM and/or indicate the presence of subclinical neuronal damage.

## Materials and methods

### Ethical considerations

This study initially used data collected during routine clinical care which was then anonymised and was thus exempt from National Health Service Research Ethics Committee (REC) review. Following initial presentation at an international conference, these findings were incorporated into routine evaluation by the clinical team. Subsequently, samples from a subset of the same cohort who had, after giving written informed consent, donated plasma samples to the Communicable Diseases Research Tissue Bank, National Research Ethics Service reference 20/SC/0226 were analysed for neurofilament-light. The consent included linkage of clinical data with the research findings.

### Study participants

Anonymised datasets of HTLV-1 ACs and patients with HAM attending the HTLV clinic at the National Centre for Human Retrovirology (NCHR), St Mary’s Hospital, London, UK, were analysed between February and March 2016. HTLV-1 infection was diagnosed by the detection of antibodies to HTLV-1 using enzyme-linked immunoassay (ELISA) or chemiluminescence (CMIA), followed by confirmation using western blot and/or the detection of HTLV-1 proviral DNA in PBMCs by polymerase chain reaction (PCR).

#### Inclusion criteria

Patients were included if they were HTLV-1 infected and either ACs (defined as HTLV-1 seropositive individuals without symptoms or signs of HAM, ATLL or other HTLV-1-associated diseases) or patients with HAM (meeting the World Health Organisation HAM diagnostic criteria (World Health Organization 1989) and had a complete dataset of HTLV-1 PVL, β2M plasma concentration and TCA markers (CD4/25, CD4/HLA-DR, CD8/25 and CD8/HLA-DR available for analysis.

#### Exclusion criteria

Patients were excluded if they were co-infected with another blood borne virus, had chronic renal disease or were on immunosuppressive therapies. Those with missing clinical and laboratory data were also excluded.

### Demographics, clinical and laboratory data

Demographics including gender, age and race and clinical status were retrieved from clinical records and exported anonymised to the study database. HTLV-1 PVL, β_2_M and TCA markers (CD4/CD25, CD4/HLA-DR, CD8/CD25 and CD8/HLA-DR — defined as percentage CD4 + or CD8 + T cells expressing surface CD25 or HLA-DR) are measured as part of the clinical routine of patients attending the NCHR. HTLV-1 PVL was quantified using real-time PCR targeting HTLV-1 tax gene and β globin gene (internal control), as previously described (Demontis et al. 2012) and TCA markers by flow cytometry using a Navios flow cytometer (Beckman Coulter, Ireland) with the following conjugated antibodies (CD45-FITC CD3-PC-5, CD4-PE, CD8-ECD, CD25- PC7 and HLA-DR APC, Beckman Coulter, UK) according to the manufacturer’s instructions. Βeta-2-microglobulin concentration in plasma was measured by Abbott Architect according to their instruction.

PVL testing was introduced in the clinical follow-up of this cohort in 1993, β_2_M in 2006 and TCA in 2011. The earliest TCA results for each patient were used with the associated PVL and β_2_M. Where data was missing (*n* = 1), the β_2_M value from the subsequent visit was used. All diagnostic results were provided by the accredited pathology laboratories of Imperial College Healthcare NHS Trust and Imperial College London.

Each investigation is performed at least annually enabling the stability of these markers over time to be determined, and thus the likely usefulness to predict disease, to be ascertained. All datasets from AC with three or more measures over a minimum of 12 months were included.

Concentration of neurofilament light (NfL) was measured in a subset of randomly selected patients (*n* = 20 HAM, *n* = 18 ACs) identified by the clinical team, who had donated plasma to the Communicable Disease Research Tissue Bank, using Single molecule array (Simoa) at University College of London (UCL), according to the manufacturer’s instructions (Quanterix, Billerica, MA).

### Statistical analysis

RStudio Desktop 0.99.896 (RStudio, Boston, MA, USA), XLSTAT 2016.2 (Addinsoft, Paris, France) and Microsoft Excel 2016 with the Analysis ToolPak Add-In (Microsoft, Redmond, WA, USA) were used to perform statistical analyses and plot graphs. Distribution differences of data between groups were tested using *p-*values generated through the Mann–Whitney *U*-test. PVL and demographic data correlations with T cell activation markers and β2-microglobulin values were examined using Spearman’s rank correlation coefficient. Chi-square test was used to verify association with the identified phenotype and the incidence of HAM during follow-up. Longitudinal data from each individual were used to calculate the slope of change (linear equation) for each marker. *p-*values less than 0.05 were considered statistically significant.

## Results

### Demographic characteristics

A total of 216 patients (158 ACs and 58 patients with HAM) were included in the study. The 158 ACs were divided into low (< 1%, *n* = 74) and high (> 1%, *n* = 84) PVL groups. Demographic data are presented in Table 1. One hundred and forty-six patients were African or Afro-Caribbean and patients with HAM were more likely to be African and Afro-Caribbean (African and Afro-Caribbean versus other races: HAM: 81% (47) vs 19% (11), ACs high PVL: 69% (58) vs 31% (26); ACs low PVL: 55.4% (41) vs 44.6% (33), p = 0.0019 (HAM vs ACs low PVL), *p* > 0.05 (HAM vs ACs high PVL and ACs low PVL vs ACs high PVL).

**Table 1:** Age at blood sample, proviral load, gender and race data for 74 low PVL and 84 high PVL HTLV-1 asymptomatic carriers, and 58 HTLV-1-associated myelopathy patients

	**Low PVL ACs**	**High PVL ACs**	**HAM patients**
	*n* = 74	*n* = 84	*n* = *58*
Mean age at blood sample (range)	44.0 (20.0–67.6)	45.5(10.2–81.9)	55.1(19.4–74.7)
Mean PVL (range)	0.28% (0.00–0.99%)	8.00% (1.01–79.6%)	15.2% (1.50–50.3%)
**Gender** *n* (%)			
Female	58 (78.4)	62 (73.8)	43 (74.1)
Male	16 (21.6)	22 (26.2)	15 (25.9)
**Race/origin** *n* (%)			
African & Afro-Caribbean	41 (55.4)	58 (69)	47 (81)
Asian	10 (13.5)	9 (10.7)	2 (3.4)
Caucasian	22 (29.7)	17 (20.3)	7 (12.2)
South American	1 (1.4)	0 (0)	2 (3.4)

Low PVL defined as < 1%, high PVL defined as > 1%

*PVL* proviral load, *HAM* HTLV-1-associated myelopathy

### T-cell activation markers correlate significantly with HTLV-1 proviral load, age and race but not with gender

Comparing the combined data of all three HTLV-1 carrier groups by Spearman’s rank correlation coefficient, CD4/25, CD4/HLA-DR, CD8/25, CD8/HLA-DR, and β_2_M all strongly and significantly correlate with PVL (Supplementary Table 1).

CD4/CD25 (*r* = 0.23, *p* = 0.001), CD4/HLA-DR (*r* = 0.31, *p* =  < 0.001), CD8/HLA-DR (*r* = 0.41, *p* =  < 0.001) and β2M (*r* = 0.31, *p* =  < 0.001) significantly correlated with patient age whereas PVL (*r* = 0.11, *p* = 0.098) and CD8/CD25 (*r* = 0.066, *p* = 0.34) had no significant correlation with age.

Given the predominance of African/Afro-Caribbeans (*n* = 146) in the cohort, this group was compared with all others combined (*n* = 70). There was a significant difference between the two race groupings for three of the TCA markers: CD4/CD25 (African/Afro-Caribbean 42.9% v 36.2%, *p* = 0.001); CD4/HLA-DR (19.0% v 14.8%, *p* = 0.008); and CD8/HLA-DR (33.9% v 28.4%, *p* = 0.023). Whilst CD8/CD25 and β2M did not differ significantly according to race (*p* > 0.05), there was a trend for PVL (8.15% vs 5.48%, *p* = 0.065). Gender did not impact on any of these markers.

### T-cell activation markers significantly differ between the three HTLV-1 patient groups (HAM, AC high viral load, AC low viral load)

**Fig. 1:**
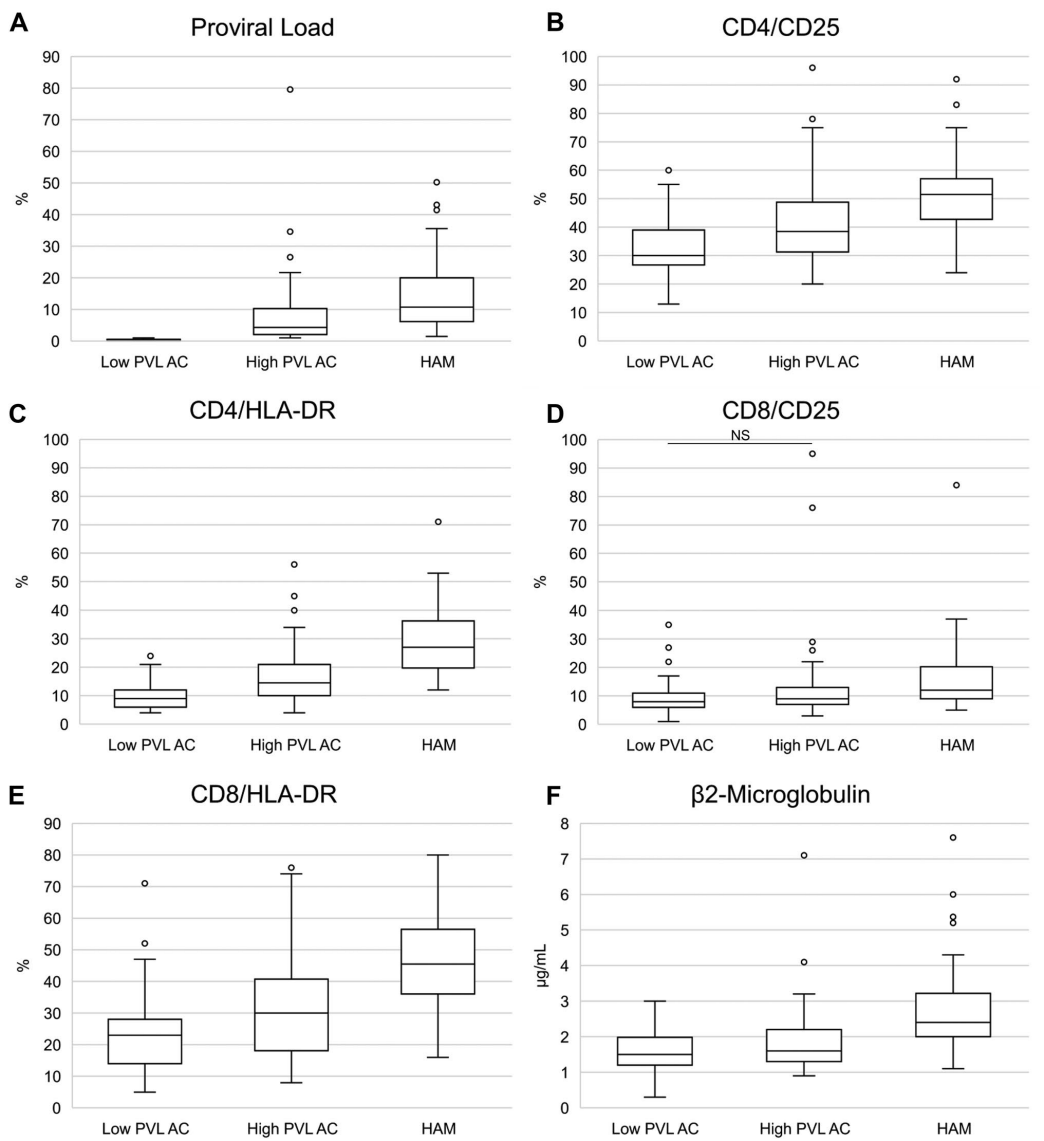
Blood test results for proviral load (PVL) **A**, T-cell activation markers **B**–**E**, and β2 microglobulin **F** in human T-lymphotropic virus type 1 asymptomatic carriers with low proviral load (PVL) (< 1%, *n* = 74) or high PVL (> 1%, *n* = 84), or patients with HTLV-1-associated myelopathy (HAM) (*n* = 58). The lower, middle and upper lines of the boxplot show the first quartile, median and third quartile values respectively. Dots represent outlier individual plasma samples (defined as beyond the first or third quartile values by ≥ 1.5 times the interquartile range). In all instances, differences between each group were significant (*p* < 0.05), except in the one comparison labelled as non-significant (NS)

HTLV-1 proviral load was significantly higher in patients with HAM (Fig. 1A) than in high proviral load ACs despite the use of ≥ 1% proviral load to distinguish high and low proviral load asymptomatic carriers. All TCA markers and β2M were significantly higher in patients with HAM than in all ACs combined. As shown in Fig. 1B–E, TCA markers and β2M (Fig. 1F) were the highest in patients with HAM and the lowest in low PVL ACs. In addition, there was a stepwise increment in the value of β2M and each T cell activation marker, except for CD8/CD25, between low and high HTLV-1 PVL ACs as well as between high PVL ACs and patients with HAM. This suggests that these markers may reflect progressive inflammation associated with HTLV-1 proviral load regardless of the clinical state (HTLV-1 PVL, TCA and β_2_M data are provided in Supplementary Table 2).

### A HAM-like phenotype identifies HAM patients within the cohort and this phenotype is shared by a small proportion of ACs

**Fig. 2:**
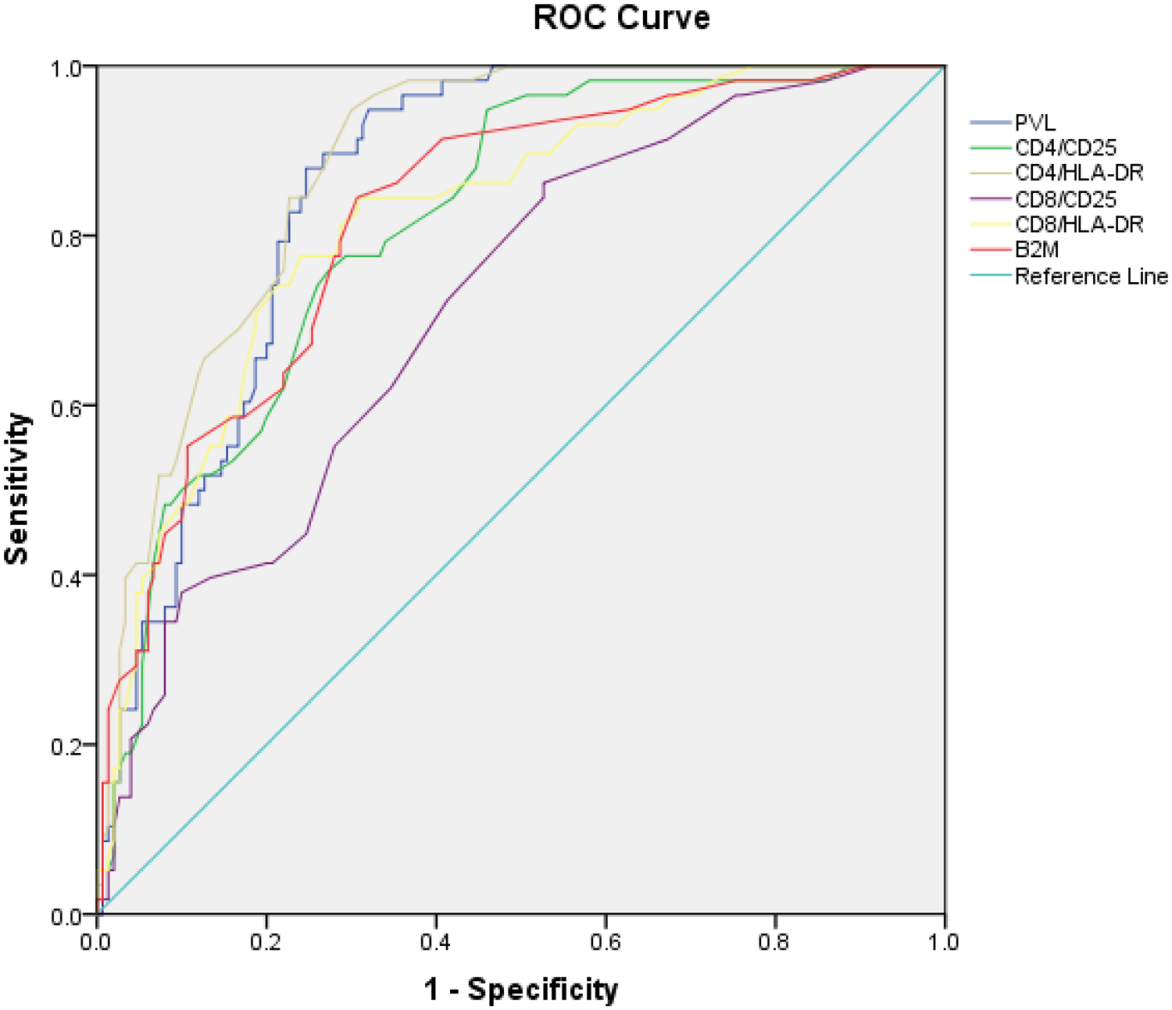
Receiver operator characteristic curves assessing sensitivity and specificity of 6 different blood tests in differentiating between HTLV-1 asymptomatic carriers (*n* = 158) and HTLV-1-associated myelopathy patients (*n* = 58)

ROC curves were generated to determine which of the markers best identify the patients with HAM out of the combined cohort of ACs and patients with HAM (*n* = 216) (Fig. 2). CD4/HLA-DR expression achieved the highest area under the curve (AUC) of 0.88, followed by HTLV-1 PVL (0.86), β_2_M (0.82), CD8/HLA-DR (0.82), CD4/CD25 (0.81) and CD8/CD25 (0.72). From these ROC curves, the cut-offs to correctly assign the highest numbers to the HAM (64%) and AC (96%) diagnoses were 15% for CD4/HLA-DR, 4.6% for PVL, 1.8 µg/mL for β_2_M, 31% for CD8/HLA-DR, 8% for CD8/25 and 35% for CD4/25. The AUC, sensitivity, specificity, positive predictive and negative predictive values for each cut-off are presented in Table 2. Each parameter was then systematically adjusted to optimise the cut-offs to identify patients with HAM rather than minimise the number of ACs assigned to HAM. As a result of this process, reducing PVL to 2.1%, CD8/CD25 to 5%, CD8/HLA-DR to 19% and β_2_M to 1.7 µg/mL increased the sensitivity of the model identifying 88% of the patients with HAM. The remaining cut-offs (CD4/CD25 ≥ 35%, CD4/HLA-DR ≥ 15%) were unchanged (Table 3). A patient was then considered having ‘HAM-like viral immune phenotype’ if it fulfilled these six criteria. Ten (6.0%) of the ACs have the same phenotype. We hypothesise that these ACs with a ‘HAM-like viral-immuno-phenotype’ are at most risk of developing HAM.

**Table 2:** Receiver operator characteristic curve analysis of the ability of proviral load (PVL), T-cell activation markers and β2 microglobulin (β2M) to differentiate between HTLV-1 asymptomatic carriers (*n* = 158) and patients with HTLV-1-associated myelopathy (*n* = 58)

			**Optimal cut-off**					
	**AUC**	**SE**	**Value**	**Youden’s J**	**Sensitivity**	**Specificity**	**PPV**	**NPV**
PVL	0.86	0.025	4.62%	0.63	88.8%	75.3%	56.7%	94.4%
CD4/CD25	0.81	0.031	35.5%	0.49	94.8%	54.0%	43.1%	96.6%
CD4/HLADR	0.88	0.022	15.5%	0.65	94.8%	70.0%	53.7%	97.4%
CD8/CD25	0.72	0.038	8.3%	0.34	86.2%	47.3%	37.5%	90.3%
CD8/HLADR	0.82	0.031	31.4%	0.54	84.5%	69.3%	50.3%	92.4%
β2M	0.82	0.031	1.85 μg/mL	0.54	84.5%	69.3%	50.3%	92.4%

*AUC* area under the curve, *SE* standard error of the AUC. Optimal cut-off, best cut-off value for each test identified by the ROC curve. *PPV* positive predictive value, *NPV* negative predictive value

**Table 3:** Viral-immuno-phenotypes to identify patients with HAM

	**Identifies 62% HAM (36) patients and 4% ACs (7)**	**Identifies 88% HAM (51) patients and 6% ACs (10)**
PVL	4.6%	2.1%
CD4/CD25	35%	35%
CD4/HLA-DR	15%	15%
CD8/CD25	8%	5%
CD8/HLA-DR	31%	19%
β2M	1.8 µg/mL	1.7 µg/mL

### β_2_M and TCAs in AC are stable over time

**Fig. 3:**
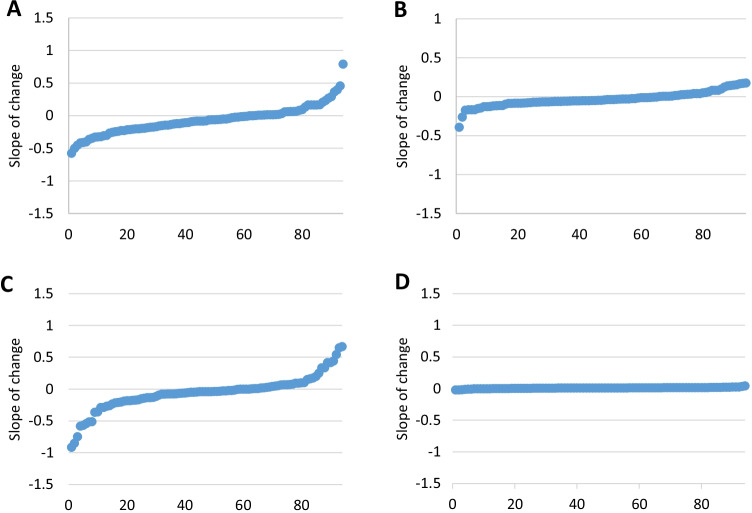
Slope of change in each of three TCA markers and in β2M in asymptomatic carriers over five years. **A** Change in CD4 CD25 expression, **B** change in CD4 HLA-DR expression, **C** change in CD8 HLA-DR expression and **D** change in plasm β2M concentration

Ninety-four asymptomatic patients were included in the longitudinal analysis. The maximum follow-up period was 60 months. There was no major trend for TCAs and β_2_M to increase or decrease over this period with small decreases in CD25 expression on CD4 cells (median slope − 0.05), and HLA-DR expression on both CD4 (median slope − 0.02) and CD8 cells (median slope − 0.06) and a small increase in β_2_M (median slope 0.009). However, as illustrated in Fig. 3, small numbers of ACs had either significantly positive or negative slopes (Fig. 3).

### NfL is increased in plasma from ACs with HAM-like phenotype at concentrations similar to patients with HAM

**Fig. 4:**
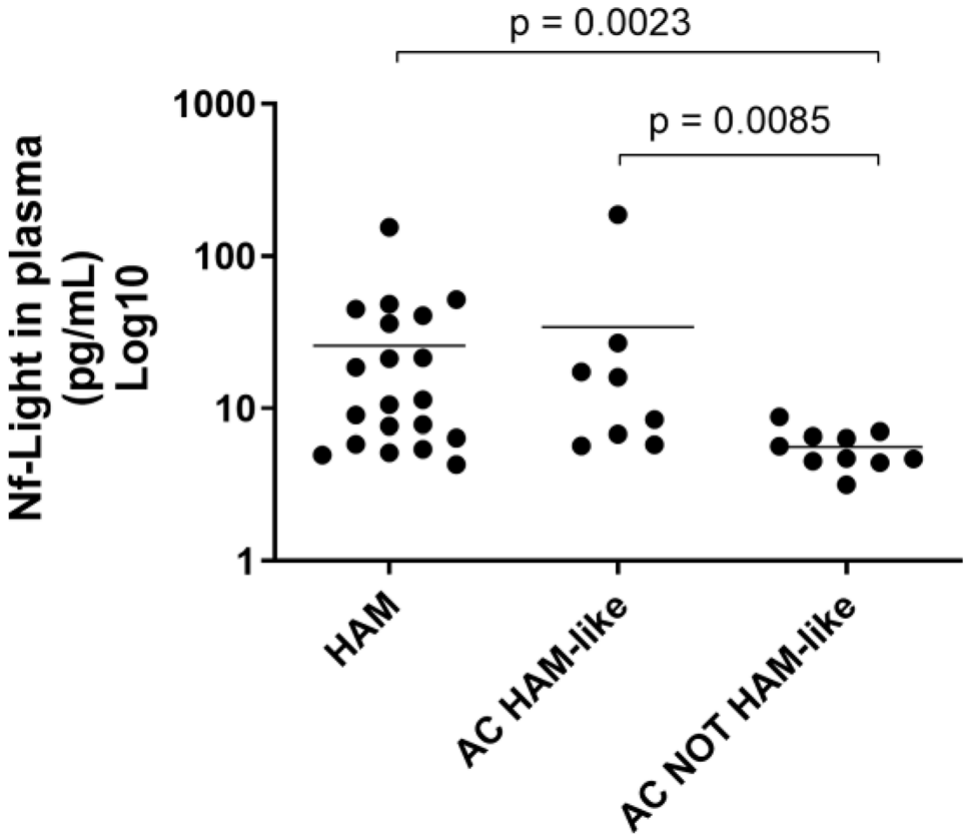
Comparison of neurofilament light in plasma of patients with HAM, patients with asymptomatic infection with HAM-like phenotype and asymptomatic individuals infected by HTLV1 without HAM-like phenotype. NfL was measured by SIMOA. Each dot represents the average of duplicates of one individual. Horizontal bars represent median. Groups were compared using Mann Whitney test. *p* < 0.05 are shown

NfL in plasma was higher in patients with HAM (*n* = 20) and ACs with HAM-like phenotype (*n* = 8) compared to ACs without HAM-like phenotype (*n* = 10) (*p* < 0.01). There was no statistically significant difference between plasma concentrations of NfL between HAM and AC with HAM-like phenotype (*p* = 0.94). There was no difference between gender and ethnicity between participants in the groups. Patients without HAM-like phenotype had similar age compared to those with HAM. Patients with HAM-like phenotype were older (median (range): HAM: 53 years (27–78); AC HAM-like = 62 years (52–76); AC not HAM-like = 55.5 years (32–65)). Nf-L in plasma did not correlate with age at sampling, except when evaluating AC without HAM-like phenotype. In this group, there was a positive correlation between NfL in plasma and age (*r* = 0.7, *p* = 0.028), as expected for the general population (Fig. 4).

### Incident HAM was restricted to ACs with a ‘HAM-like’ viral-immuno-phenotype

Since the identification of this putative high-risk (HAM-like) phenotype in 2017, two asymptomatic carriers whose data were included in the study have developed HTLV-1-associated myelopathy. In both cases, their viral-immuno-phenotype met the ‘HAM-like’ criteria described above prior to the development of HAM. Thus 2/10 with the ‘HAM-like’ phenotype have developed HAM whereas none of the 148 carriers without this phenotype have thus far developed myelopathy (chi-square *p* = 0.0036).

## Discussion

The present study identified markers that differentiate patients with HAM from HTLV-1 ACs, but more importantly, it shows that the proposed markers can identify a proportion of ACs with high risk of developing HAM. The hypothesis was strengthened by the increased levels of NfL observed in that group. Moreover, during follow-up, incident cases of HAM arose only amongst those ACs identified as high risk according to the proposed markers. This is also one of few studies to examine an AC cohort split into HAM-risk categories by PVL, or to make clinical recommendations for the use of TCA markers in HTLV-1 ACs.

Patients with HAM have significantly increased CD25 and HLA-DR expression on both CD4 + and CD8 + T cells compared with ACs. Corroboratively, a higher percentage of CD4 + HLA-DR + T-cells has been reported in patients with HAM compared with ACs (Brito-Melo et al. 2002). Furthermore, ACs with high PVL were also found to have significantly higher expression of all but one TCA marker than low PVL ACs, elaborating on a previous small study (*n* = 20) which observed non-significantly higher CD4/CD25, CD4/HLA-DR and CD8/CD25 expression in ACs with PVL > 1% compared with ACs with < 1% PVL (Coutinho et al. 2014). Combined, these studies show that both disease expression and raised PVL are associated with enhanced T cell activation in HTLV-1 infection. Plasma concentrations of β_2_M have previously been shown to be significantly higher in HAM patients than ACs, and to correlate with HAM disease progression (Kirk et al. 2011; Toledo-Cornell et al. 2014). This study supports this finding revealing also that the elevation in β_2_M in patients with HAM is at least in part independent of their high proviral load.

Analysis revealed many significant correlations, most importantly that increasing PVL correlates with increasing TCA markers, substantiating the earlier evidence that higher PVL (and by extension higher HAM risk) is associated with increased immune activation (Coutinho et al. 2014). Furthermore, despite both correlating strongly, the r_s_ value for the two CD4 + T cell activation markers was nearly double that of the CD8 + T cell activation markers (0.58 and 0.63 *v* 0.35 and 0.39) (Supplementary Table 2), highlighting the importance of CD4 + T cells in HAM pathophysiology where the role of the CD8 response has often been emphasised. Also of interest are relationships with race, with comparisons possible due to the UK HTLV-1 carrier cohort’s diverse composition. The lifetime HAM risk has been estimated at 0.3% in a Japanese cohort (Kaplan et al. 1990) but six times higher, at 1.9%, in a mixed Jamaican and Trinidadian cohort (Maloney et al. 1998). The reason for this difference is unknown, with Gessain & Cassar asserting that these groups are infected with the same HTLV-1 subtype A, suggesting that the difference is possibly due to differing immunological responses. During 8 years follow-up, up to 30% of asymptomatic patients developed neurological symptoms or signs in Brazil (Tanajura et al. 2015), whereas mild and subclinical manifestations of HAM were also observed in 24% of asymptomatic carriers in a different cohort in the same country (Haziot et al. 2019). The present study reveals African & Afro-Caribbean race to be significantly, albeit weakly, associated with TCA markers, in addition to a trend to having higher PVL. This may partially explain the reported geographical variation in estimated lifetime HAM-risks, although a full immunological explanation is beyond the scope of this study. A complex mixture of genetic and environmental factors also likely contributes to HAM. HLA-DRB1*0101 for example has already been associated with raised HAM risk, and comparatively studied in Iranian and Japanese ethnicities (Sabouri et al. 2005). One limitation is that HAM may be under-diagnosed amongst Caucasians in the UK population compared with those of Afro-Caribbean origin leading to over-representation of the high proviral load carriers of Afro-Caribbeans in our cohort. Environmental factors may also alter HAM susceptibility, such as HIV-1 co-infection, which may increase the risk of HAM although the evidence is inconclusive (Casseb et al. 2007). Comment is not possible on, the frequently studied, South Americans due to their underrepresentation in the UK cohort. Additionally, increasing PVL and T-cell activation is found to be associated with increasing age, offering insight into the propensity for HAM to present during middle age (Maloney et al. 1998).

Creation of ROC curves allowed calculation of optimal cut-off points for detecting HAM patients out of the combined AC and HAM patient groups. Difficulty in ascertaining an optimal PVL cut-off for categorising ACs as low or high HAM risk has historically troubled the HTLV-1 research community, with the commonly used value being 1% (Taylor et al. 1999; Demontis et al. 2012). Gonçalves et al. additionally proposed 1% as a cut-off point between low and intermediate HAM risk, and 5% as a cut-off between intermediate and high HAM risk (Gonçalves et al. 2008). The present study found the optimal cut-off between low and high HAM risk to be towards the lower end of these previous proposals, at 2.1%.

The optimal calculated cut-offs for TCA markers and β_2_M make sensible values for HAM risk prediction, despite, surprisingly, being mostly within our clinic’s reference ranges, representing the lower predictive power of individual TCA markers. It is therefore clinically crucial to consider all four TCA markers and β_2_M both in unison, and within the context of the patient’s PVL. It should be noted that with the exception of β_2_M, the assays are not commercial kits and will be subject to variation between laboratories. Centres measuring HTLV-1 proviral load and using flow cytometry to quantify T-cell activation would therefore need to establish their own cut-offs either from existing cohorts or inter-laboratory comparison.

Despite PVL being shown to remain stable over several years (Demontis et al. 2012; Kwaan et al. 2006; Olavarria et al. 2012), no previous research has tracked stability of HTLV-1 carriers’ TCA markers and β_2_M over time. Since TCA measures were introduced as part of routine clinical care in 2011, longitudinal changes could be assessed in the same cohort for a maximum of 5 years. These were remarkably stable suggesting that, as with HTLV-1 proviral load, an individual’s propensity to develop HAM may be determined early in infection and thus is potentially predictable. Consequently, the selection of 6% of the AC group with a HAM-like profile led to the hypothesis that these carriers would be at most risk of developing HAM.

To confirm the hypothesis that a HAM-like phenotype predates and predicts HAM would require longitudinal study of a cohort, with incident cases of HAM, in which all these parameters were known or could be retrospectively assessed. Having identified the HAM-like phenotype in 2016, the clinic database was reviewed after a further 4 years of follow-up. Two carriers had developed HAM. Both had a HAM-like profile that predated clinical symptoms or signs. The probability of this association being due to chance was 4 in 1000.

Subsequently, we sought evidence of subclinical neuronal damage and have shown that ACs with HAM-like phenotype have higher concentrations of NfL when compared to AC without this phenotype. Moreover, NfL in “HAM-like ACs” was comparable to HAM. Our group recently showed that plasma concentrations of NfL strongly correlate with NfL in cerebrospinal fluid (CSF) and with markers of central nervous system (CNS) inflammation, such as neopterin and CXCL10 in CSF in patients with HAM. We also demonstrated that NfL is useful to determine HAM severity (Rosadas et al. 2021). Here, we show evidence that those patients with the HAM-like phenotype have subclinical neuronal damage which lends support to the hypothesis that they are at high risk of developing HAM.

In conclusion, TCA and β_2_M have been demonstrated to be significantly different between low PVL ACs, high PVL ACs and HAM patients. These findings reinforce and expand upon the current understanding of HAM pathophysiology. Additionally, previous PVL cut-offs for predicting an AC’s HAM risk have been tested and refined, alongside the introduction of evidenced TCA marker and β_2_M HAM-risk cut-offs. The validity of a hypothesised HAM-like profile has been supported by the incident cases during a period of 4 years and increased concentrations of NfL in plasma. We hope that the ability to identify ACs for targeted follow up will improve earlier diagnosis, management and treatment of HAM, and anticipate that this will lead to better outcomes for affected patients.

## Supplementary Information

Below is the link to the electronic supplementary material.

Supplementary file1 (PDF 77 KB)

Supplementary file2 (PDF 151 KB)
