# Supplementary material for: Refining the risk of HTLV-1-associated myelopathy in people living with HTLV-1: identification of a HAM-like phenotype in a proportion of asymptomatic carriers
Source: J Neurovirol. 2022 Jul 30;28(4-6):473–82. doi: 10.1007/s13365-022-01088-x (PMC9797460; doi:10.1007/s13365-022-01088-x)
Supplement: Supplementary file 1 — Supplementary file1 (PDF 77 KB) [file 13365_2022_1088_MOESM1_ESM.pdf]

**Supplementary Table 1.** Spearman's rank correlation coefficients analysing relationships between T cell activation markers, B2M and HTLV-1 Proviral Load (PVL).

| | **PVL** | |
|---|---|---|
| | **r** | **P value** |
| PVL % | - | - |
| CD4/25 % | 0.58 | **<0.001** |
| CD4/HLADR % | 0.63 | **<0.001** |
| CD8/25 % | 0.35 | **<0.001** |
| CD8/HLADR % | 0.39 | **<0.001** |
| β2M μg/mL | 0.35 | **<0.001** |
