# Supplementary material for: Refining the risk of HTLV-1-associated myelopathy in people living with HTLV-1: identification of a HAM-like phenotype in a proportion of asymptomatic carriers
Source: J Neurovirol. 2022 Jul 30;28(4-6):473–82. doi: 10.1007/s13365-022-01088-x (PMC9797460; doi:10.1007/s13365-022-01088-x)
Supplement: Supplementary file 2 — Supplementary file2 (PDF 151 KB) [file 13365_2022_1088_MOESM2_ESM.pdf]

Supplementary Table 2 - Statistical analysis of proviral load (PVL), T-cell activation markers, and β_2 microglobulin (β2M) distribution amongst different HTLV-1 carrier groups.

	PVL %	CD4/CD25 %	CD4/HLA-DR %	CD8/CD25 %	CD8/HLA-DR %	β2M μg/mL
**Low PVL ACs**						
Mean	0.3	32.1	9.5	9.5	22.6	1.6
Median	0.1	30.0	9.0	8.0	23.0	1.5
SD	0.3	8.9	4.2	5.6	11.7	0.5
Lowest	0.0	13.0	4.0	1.0	5.0	0.3
Highest	1.0	60.0	24.0	35.0	71.0	3.0
**High PVL ACs**						
Mean	8.0	40.6	16.7	12.0	30.6	1.8
Median	4.4	38.5	14.5	9.0	30.0	1.6
SD	10.7	13.5	9.6	12.6	14.8	0.8
Lowest	1.0	20.0	4.0	3.0	8.0	0.3
Highest	79.6	96.0	56.0	95.0	76.0	7.1
**Combined ACs**						
Mean	4.4	36.6	13.3	10.8	26.8	1.7
Median	1.2	34.5	11.0	9.0	25.0	1.6
SD	8.7	12.3	8.4	10.0	14.0	0.7
Lowest	0.0	13.0	4.0	1.0	5.0	0.3
Highest	79.6	96.0	56.0	95.0	76.0	7.1
**HAM Patients**						
Mean	15.2	51.9	29.3	16.5	46.6	2.8
Median	10.8	51.5	27.0	12.0	45.5	2.4
SD	12.5	13.7	12.6	12.2	15.9	1.3
Lowest	1.5	24.0	12.0	5.0	16.0	1.1
Highest	50.3	92.0	71.0	84.0	80.0	7.6
**P Values**						
Low PVL ACs vs High PVL ACs	**<0.001**	**<0.001**	**<0.001**	0.10	**<0.001**	**0.026**
Low PVL ACs vs HAM Patients	**<0.001**	**<0.001**	**<0.001**	**<0.001**	**<0.001**	**<0.001**
High PVL ACs vs HAM Patients	**<0.001**	**<0.001**	**<0.001**	**0.036**	**<0.001**	**<0.001**
Combined ACs vs HAM Patients	**<0.001**	**<0.001**	**<0.001**	**0.002**	**<0.001**	**<0.001**

HTLV-1 carrier groups include low PVL asymptomatic carriers (ACs, <1%, n=74), high PVL ACs (>1%, n=84), combined AC groups (n=158), and patients with HTLV-1-associated myelopathy (HAM, n=58). SD: standard deviation values. Lowest and highest: lowest and highest recorded values in each group. Units for each test: PVL, CD4/25, CD4/HLA-DR, CD8/25, CD8/HLA-DR - %; β_2 microglobulin – mg/L. **Bold** p-values indicate significance ($p<0.05$).
